# Fecal microbiota composition is linked to the postoperative disease course in patients with Crohn’s disease

**DOI:** 10.1186/s12876-020-01281-4

**Published:** 2020-05-04

**Authors:** Anna Strömbeck, Anders Lasson, Hans Strid, Johanna Sundin, Per-Ove Stotzer, Magnus Simrén, Maria K. Magnusson, Lena Öhman

**Affiliations:** 1 Department of Microbiology and Immunology, Institute of Biomedicine, Sahlgrenska Academy, University of Gothenburg, Box 435, 405 30 Gothenburg, Sweden; 2 Department of Internal Medicine, Södra Älvsborg Hospital, Borås, Sweden; 3 Department of Internal Medicine, Sahlgrenska University Hospital, Gothenburg, Sweden; 4 Department of Internal Medicine and Clinical Nutrition, Institute of Medicine, Sahlgrenska Academy, University of Gothenburg, Gothenburg, Sweden

**Keywords:** Crohn’s disease, Fecal microbiota, Postoperative disease recurrence

## Abstract

**Background:**

The role of the fecal microbiota composition for the postoperative disease course of patients with Crohn’s disease (CD) who have undergone ileocecal resection remains to be established. In this study, we investigated if the fecal microbiota composition, determined by a high throughput test quantifying a pre-selected set of bacteria, is associated with the postoperative disease course of CD patients.

**Methods:**

Fecal samples were obtained from healthy subjects as well as from CD patients, 3–10 weeks and 1 year after ileocaecal resection. The fecal microbial composition was analyzed by Genetic Analysis GA-map Dysbiosis test, targeting ≥300 bacteria on different taxonomic levels. Postoperative disease status was assessed endoscopically according to Rutgeerts scoring system 1 year after surgery. Differences in fecal microbiota composition between groups were analyzed by multivariate factor analyses and cluster analysis. Microbial stability over time was determined using Bray-Curtis dissimilarity.

**Results:**

One year after surgery, the fecal microbiota composition differed between CD patients (*n* = 21) and healthy subjects (*n* = 7). At this time point, the microbiota composition of CD patients was associated with disease course, clearly separating patients with disease relapse (*n* = 8) and patients in remission (*n* = 13). Further, the microbial within-patient stability was high during the first year after surgery, irrespective of disease course.

**Conclusion:**

The fecal microbiota composition of CD patients, analyzed by GA-map Dysbiosis test, is subject to little variation over time, and may potentially be used as a non-invasive diagnostic tool for the postoperative disease course.

## Background

Despite increased use of immunosuppressive and anti-tumour necrosis factor drugs, approximately 25% of adult patients with Crohn’s disease (CD) are in need of surgery within 10 years of diagnosis [1, 2]. The most common type of surgical procedure for patients that are refractory to medical treatment or develop complications is ileocaecal resection. However, surgical resection is not curative, and approximately 30% of all patients experience postoperative disease recurrence within 5 years of surgery [3–5]. It is not yet known why many patients attain long-term remission while others suffer from an early disease recurrence, and there are currently no reliable non-invasive tools to predict disease postoperative disease course.

Numerous studies have documented differences in gut microbial profiles between patients with CD and healthy individuals, with a reduced microbial diversity and a lower relative abundance of specific bacteria in CD patients [6–11]. The relationship between gut microbiota profile and CD has further been accentuated by recent studies suggesting that fecal microbiota transplantation from healthy donors may be beneficial for CD patients [12–14]. Efforts have been made to evaluate the link between microbiota of mucosal biopsies and the postoperative disease course of the patients. These studies indicate that distinct profiles of mucosa-associated microbiota at the time of surgery and postoperatively are associated with disease recurrence and remission, respectively, and that these profiles can be distinguished from each other and from the microbiota profiles of healthy individuals [15–21]. However, to the best of our knowledge only two previous studies have addressed the relationship between fecal microbiota composition and postsurgery endoscopic status, although with inconclusive results [7, 15].

The clinical use of previous findings have so far been limited as sequencing microbiome regions of 16 s rRNA with subsequent phylogenetic analysis is a cumbersome task, requiring advanced equipment and in-depth knowledge of data computation and microbiota. Potentially, user-friendly tools, simplifying the analysis of the fecal microbiota, may facilitate the utilization of microbiota profiling in a clinical setting to predict the postoperative disease course in CD. The aim of this study was therefore to determine if the fecal microbiota composition, determined by a high throughput and easy-to-use test, quantifying a pre-selected set of bacteria, is associated with the postoperative disease status of CD patients.

## Methods

### Study subjects and sample collection

Adult patients, 18–63 years, who had undergone ileocaecal resection for CD, were recruited from four gastroenterology units in southwest Sweden from September 2008 to December 2011 [22]. As previously described [22], exclusion criteria were disease activity beyond the ileocaecal region, malignancy during the last 5 years, severe chronic disease affecting the ability to comply with the study protocol (i.e., renal or liver failure, severe cardiovascular disease, neurological disease, and psychiatric disorder), use of non-steroidal anti-inflammatory drugs (5-aminosalicylic acid not included), drug addiction, and pregnancy at inclusion. All patients were given a single dose of perioperative antibiotic prophylaxis. None of the patients had previously undergone a bowel resection.

Approximately 1 year after surgery, an ileocolonoscopy was performed and the mucosa in neoterminal ileum and ileocolonic anastomosis was assessed according to Rutgeerts scoring system [23]. Five or less aphthoid lesions (i_0_-i_1_) were considered as remission and more than five aphthoid lesions, lesions confined to the anastomosis or diffuse inflammation (i_2_-i_4_) were considered as endoscopic disease recurrence.

Fecal samples from CD-patients were collected from the first bowel movement in the morning 2 weeks prior to or 1–4 weeks after the ileocolonoscopy at the follow up 1 year after surgery, and for some patients also at 3–10 weeks after the ileocaecal resection. Fecal samples were stored at − 20 °C until analysis.

Fecal samples were also collected from healthy subjects recruited at Sahlgrenska University hospital, Gothenburg, and stored at − 80 °C until analysis. None of the healthy subjects had any history of gastrointestinal or other chronic disorders or had taken any immunosuppressive agents, antibiotics or any other medications during the last 3 months prior to sample collection.

### Microbiota analysis

Fecal microbiota was analyzed by the GA-map Dysbiosis test (Genetic Analysis AS, Oslo, Norway) as previously described [24]. In brief, the test is based on molecular biology techniques whereby bacterial probes, based on the 16S rRNA sequence in seven variable regions (V3–V9), measure the abundance of bacteria according to the strength of fluorescent signal detection (probe intensity). The GA-map test comprises 54 DNA probes targeting ≥300 bacteria on different taxonomic levels [25]. All probes are listed in Supplementary Table 1.

### Data analyses

Data analyses for principal component analysis (PCA), Bray-Curtis dissimilarity and clustering were performed in R Studio 1.1.456 (R 3.6.1) using pca3d and vegan packages. Bray-Curtis dissimilarity was used to investigate differences within and between study subjects at different time points. A dissimilarity matrix was calculated using all samples from both time points in the same model. Within-patient analysis over time shows dissimilarities between early and 1-year follow up for the same patients. Between-patient analysis over time show mean dissimilarities between early samples and all non-related samples at 1-year follow up. Between-patient analysis at 1-year follow up show mean dissimilarities between unique patients and all non-related samples (at the same time point). Lower numbers define higher similarity between samples. For post hoc analysis, Kruskal-Wallis test followed by *Dunn’s* multiple comparisons test was used (GraphPad Prism; GraphPad Software, La Jolla, CA, USA). Cross-tabulation using Fisher’s Exact Test and Spearman correlation analysis were performed in IBM SPSS Statistics version 25.

To examine the difference in fecal microbial composition (X-variables) between CD patients and healthy subjects, as well as between CD patients in remission and with postoperative disease recurrence (Y-variables), multivariate factor analysis was used (SIMCA 15 software; Umetrics, Umeå, Sweden). As previously described [24], orthogonal partial least squares discriminant analysis (OPLS-DA) was employed to correlate X-variables with a selected Y-variable and with each other in linear multivariate models. The quality of OPLS-DA is based on the parameter R2, i.e. the goodness of fit of the model (values ≥0.5 define good discrimination, best possible fit R2 = 1), and Q2, i.e., the goodness of prediction of the model (values ≥0.5 define high predictive ability). In the OPLS-DA loadings column plots and scatter plots, each X variable is shown in relation to Y. The X-variables positioned furthest to the left or right are more closely related to the respective Y variable, and the stronger and more reliable are their contribution to the model. A variable influence on projection (VIP) was used as a variable selection based on discriminatory power since the number of variables exceeded the number of study subjects. To reduce the risk of over fit, post hoc 100 permutation tests of OPLS-DA models were performed and only models with permutation indices fulfilling the post hoc analysis criteria of R ≤ 0.4 and Q2Y < 0.05 were accepted [26]. Differences in bacterial abundance between two groups were further analyzed with Mann-Whitney U test (GraphPad Prism). Statistically significant differences are indicated with asterisks in the OPLS-DA loadings column plots (* *p* ≤ 0.05; ** *p* ≤ 0.01; *** *p* ≤ 0.001; **** *p* ≤ 0.0001).

## Results

### Clinical and demographic characteristics of the study subjects

In total, 21 patients were included in the study. Clinical and demographic characteristics for the patients are shown in Table 1. Patients were classed based on disease behavior according to the Montreal classification system (B1-B3). In total, 2 patients had surgery performed due to inflammatory disease behavior (B1), 14 patients due to stricturing disease (B2), and 5 patients due to a perforating disease behavior (B3) (Table 1). To examine the small intestine, a computed tomography and/or a magnetic resonance enterography or small-bowel series was performed in a majority of the patients prior to surgery or at postoperative follow up, as previously described [22]. At surgery, a careful examination of the small intestine was performed in all patients.

**Table 1:** Demographic and clinical characteristics of the patients

	*n = 21*	
Characteristics	*n*	(%)
Sex		
Female/Male	7/14	(33/67)
Median age (at inclusion)		
28 years (17–63)		
Median disease duration		
3 years (0–11 years)		
Smoking (at inclusion)		
Never	12	(57)
Past	6	(29)
Present	3	(14)
Disease behavior		
Inflammatory	2	(10)
Stricturing	14	(67)
Perforating	5	(24)
Therapy at time of resection		
No medication	4	(19)
5-ASA^a^	7	(33)
Corticosteroids	13	(62)
Thiopurines	10	(48)
Anti-TNF^b^	1	(5)
Therapy at time of colonoscopy		
No medication	8	(38)
5-ASA	8	(38)
Corticosteroids	2	(10)
Thiopurines	6	(29)

^a^5-aminosalicylic acid

^b^Anti- tumor necrosis factor

**Fig. 1:**
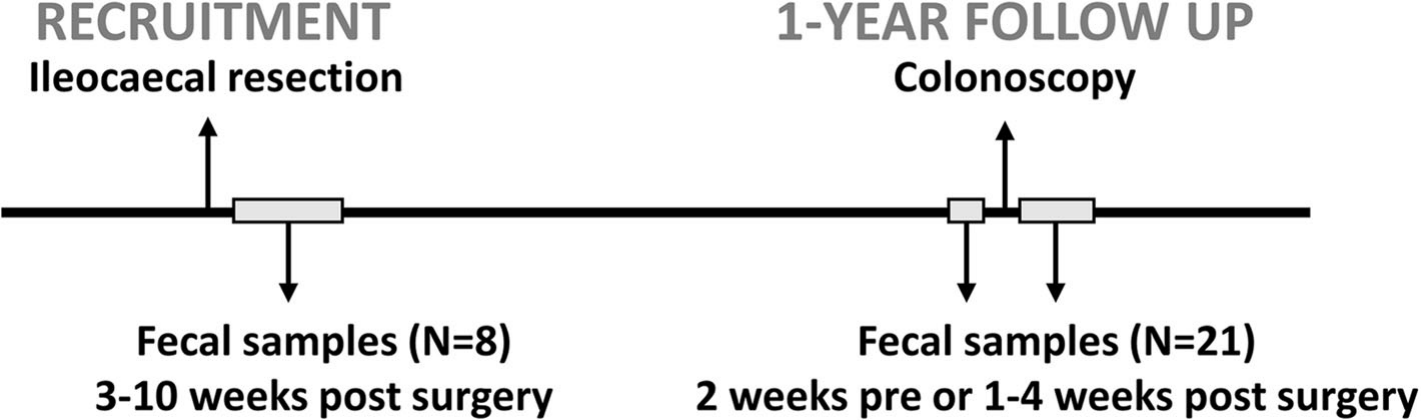
Graphical flowchart of the prospective study design. Adult patients who had undergone ileocaecal resection for Crohn’s disease were included (*N* = 21). Fecal samples were obtained 3–10 weeks after resection (*N* = 8) and 1 year after resection (*N* = 21). Postoperative disease status was assessed by colonoscopy approximately 1 year after surgery

Steroid treatment in conjunction to surgery was quickly de-escalted postoperatively, and introduction of any new medical treatments postoperatively, including corticosteroids, was effected on clinical basis throughout the whole follow-up period until the colonoscopy 1 year after surgery. Ileocolonoscopy was performed at follow up, a median time of 52 weeks (41–58 weeks) after ileocaecal resection, to evaluate the inflammatory activity in the colon and to assess the ileocolonic anastomosis and the neoterminal ileum according to Rutgeerts score. The colonic mucosa distal to the anastomotic area was endoscopically normal in all but one patient. This patient had a local oedema in the descendent colon, and a corresponding histological inflammation was detected. According to Rutgeerts scoring system, 13 patients (62%) were considered to be in endoscopic remission (i_0_-i_1_), whereas 8 patients (38%) had endoscopic disease recurrence (i_2_-i_4_) at 1-year follow up (Table 2). A graphical flowchart of the study design is shown in Fig. 1.

**Table 2:** Rutgeerts’ endoscopic score for recurrence of Crohn’s disease 1 year after ileocaecal resection

	*n = 21*	
Rutgeerts’score	*n*	(%)
i_0_	4	(19)
no lesions		
i_1_	9	(43)
≤ 5 aphthous lesions		
i_2_	7	(33)
> 5 aphthous lesions with normal mucosa between the lesions or skip areas of larger lesions or lesions confined to the ileocolonic anastomosis		
i_3_	1	(5)
diffuse aphthous ileitis with diffusely inflamed mucosa		
i_4_	0	(0)
diffuse inflammation with already larger ulcers, nodules, and/or narrowing

All patients provided stool samples 1 year after ileocaecal resection (termed 1-year follow up) and eight of the patients also provided samples at 3–10 weeks after ileocaecal resection (termed early follow up). In addition, stool samples were collected from seven healthy subjects (four women) with median age 25 (20–36).

### Fecal microbiota composition at 1-year follow up discriminate CD patients from healthy subjects

**Fig. 2:**
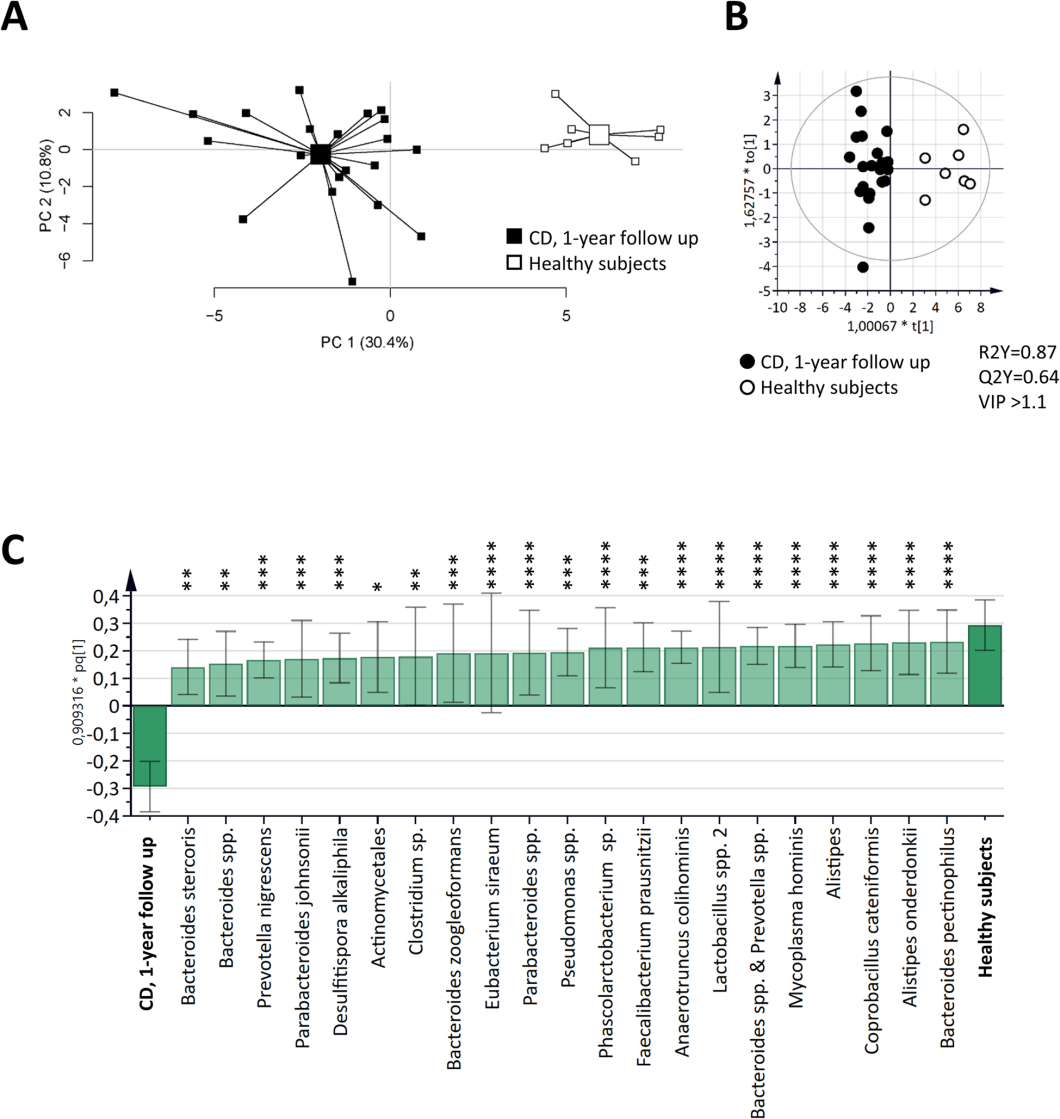
Differences in fecal microbiota composition in CD patients and healthy subjects (HS). Fecal samples from CD patients 1 year after ileocaecal resection (*n* = 21) and from HS (*n* = 7) were analyzed by the GA-map™ Dysbiosis test. **a** PCA plot of CD patients and HS based on fecal bacterial taxa (X-variables; *n* = 54). Lines to the centroid, showing the group mean, connect each group. OPLS-DA scatter plot (**b**) and loadings column plot (**c**) of CD patients and HS (Y-variables) and fecal bacterial taxa (X-variables; VIP > 1.1; *n* = 21). Statistically significant differences between study groups are indicated with asterisks in the OPLS-DA loadings column plot (* *p* ≤ 0.05; ** *p* ≤ 0.01; *** *p* ≤ 0.001; **** *p* ≤ 0.0001)

At 1-year follow up, the fecal microbiota composition defined by the GA-map Dysbiosis test differentiated CD patients from healthy subjects (Fig. 2a). A clear discrimination between CD patients and healthy subjects was also observed in the OPLS-DA score scatter plot (Fig. 2b). A majority of the bacterial taxa (31 of 54) showed higher abundance in healthy subjects as compared to CD, while only two were higher among CD patients, i.e. *Ruminococcus gnavus* and *Shigella* spp. & *Echerichia* spp. (Supplementary Table 1). The 21 most important bacteria for the discrimination between CD patients and healthy subjects, all with a higher abundance in healthy subjects, are shown in Fig. 2c.

### Fecal microbiota composition at 1-year follow up is associated with disease course

**Fig. 3:**
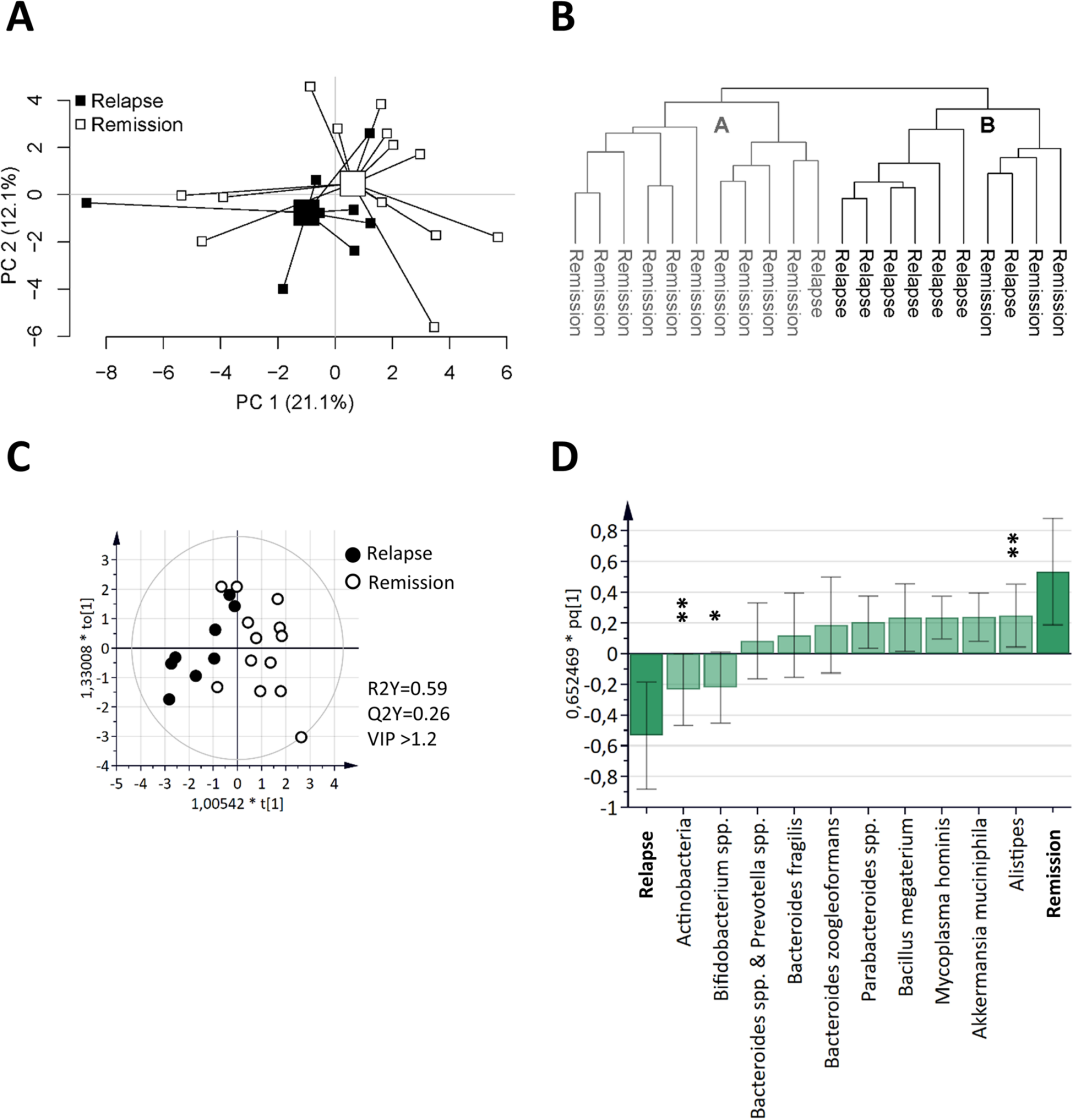
Associations between fecal microbiota and postsurgical disease course in CD. Fecal microbiota from CD patients with endoscopic disease relapse (*n* = 8) and patients with endoscopic disease remission (*n* = 13) 1 year after ileocaecal resection was analyzed by the GA-map™ Dysbiosis test. **a** PCA plot of CD patients with disease relapse and patients with remission based on fecal bacterial taxa (X-variables; *n* = 54). Lines to the centroid, showing the group mean, connect each group. **b** Cluster analysis of fecal bacterial taxa (*n* = 54) from CD patients with disease relapse and remission 1 year after ileocaecal resection (*n* = 21). OPLS-DA scatter plot (**c**) and loadings column plot (**d**) of postoperative disease course (Y-variables, i.e., disease relapse vs remission) and fecal bacterial taxa (X-variables; VIP > 1.2; *n* = 10). Statistically significant differences between study groups are indicated with asterisks in the OPLS-DA loadings column plot (* *p* ≤ 0.05; ** *p* ≤ 0.01)

Next, the fecal microbial composition in CD patients at 1-year follow up and the concurrent postoperative disease status, was examined. A separation between CD patients in endoscopic remission and with endoscopic disease recurrence was indicated by the microbiota composition using PCA (Fig. 3a). Cluster analysis based on the microbiota defined two clusters, cluster A with 11 members and cluster B with 10 members (Fig. 3b). As seen by the cross tabulated frequencies in Table 3, there was a significant relationship between the microbiota and the postoperative disease status, *p* = 0.008. The 10 most important bacterial taxa for this discrimination were identified using OPLS-DA (Fig. 3c-d), with *Alistipes* in higher abundance and Actinobacteria and Bifidobacterium spp. in lower abundance in patients in endoscopic remission as compared to patients with disease recurrence (Fig. 3d). *Alistipes*, which was decreased in CD as compared to healthy subjects (Supplementary Table 1), was also found to correlate negatively to Rutgeerts score, r = − 0.659 and *p* = 0.001.

**Table 3:** Cross-tabulation of 1-year post-operative disease status and microbiota clusters, *p* = 0.008^a^

Post-operative status	Microbiota cluster^b^		Total
	A	B	
Remission Relapse	10	3	13
	1	7	8
Total	11	10	21

^a^Fisher’s Exact Test

^b^Defined by the cluster analysis in Fig. 3b

### Fecal microbiota composition at early follow up is similar to 1-year follow up

**Fig. 4:**
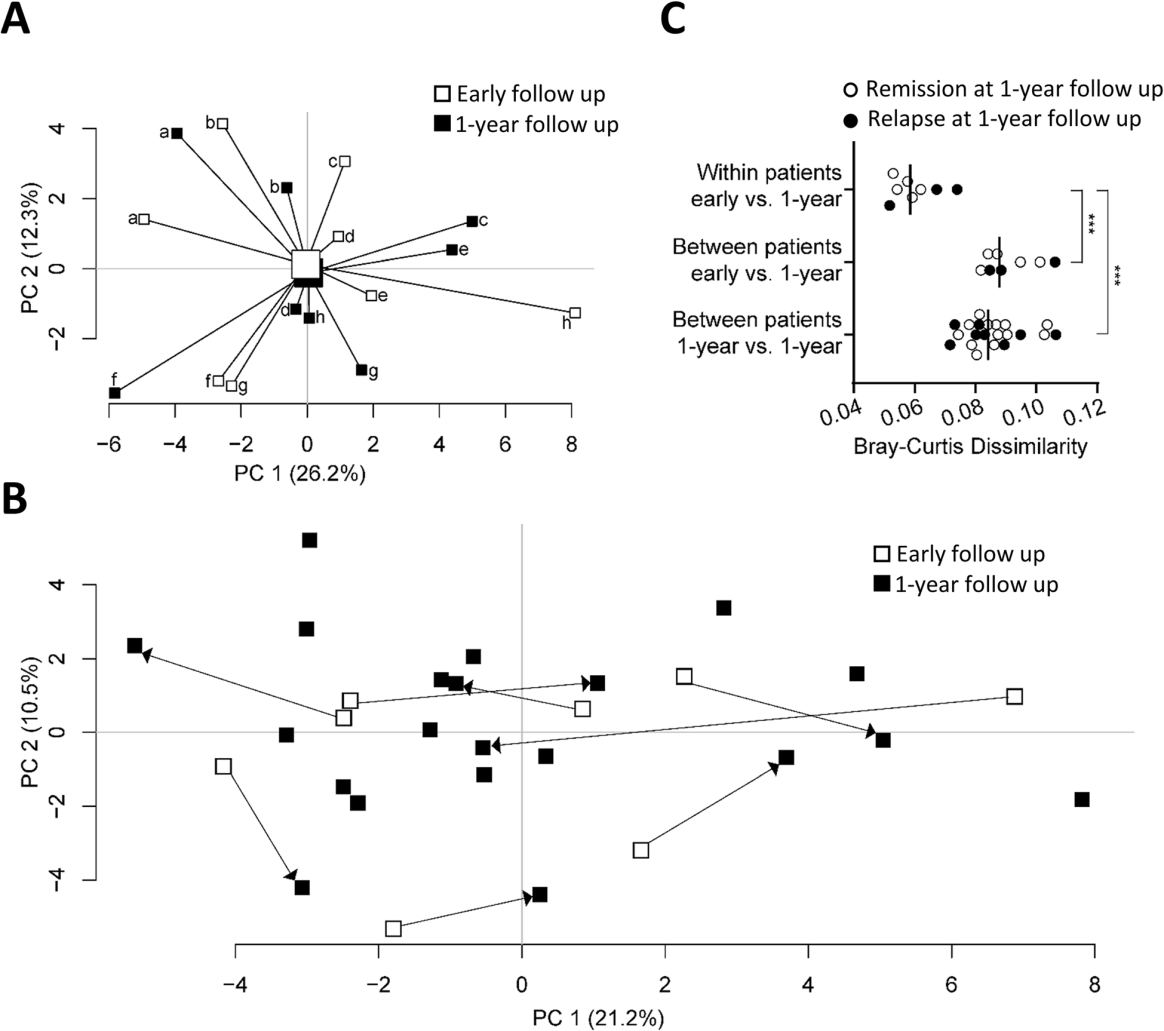
Stability of fecal microbiota composition the first year after ileocaecal resection. Fecal microbiota from 8 CD patients at early follow up (3–10 weeks after ileocaecal resection) and at the 1-year follow, and from 13 patients at the 1 year follow up was analyzed by the GA-map™ Dysbiosis test. **a** PCA based on fecal microbiota composition (X-variables; *n* = 54) from 8 CD patients at early follow up and from the same patients at the 1-year follow up. Lines to the centroid, showing the group mean, connect each group. Individual patients are marked from a-h for each time point. **b** PCA displaying the within-patient mobility of fecal microbial composition from early follow up to 1-year follow up in relation to the total population at the 1-year follow up (*n* = 21) (paired samples indicated with arrows pointing from early to 1-year follow up, *n* = 8). **c** Within-patient microbial similarity from early follow up to 1-year follow up (top, *n* = 8) in relation to between-patient similarity from early follow up to 1-year follow up (middle, *n* = 8), and to between-patient similarity at 1 year follow up (bottom, *n* = 21) analyzed by Bray-Curtis dissimilarity index. Between-patient dissimilarities show mean values for a unique individual to all non-related patients (mean of *n* = 20). Closed and open circles represent patients with disease relapse and remission, respectively, at the 1-year postsurgical follow-up; *** *p* ≤ 0.001

Finally, we examined whether the fecal microbiota composition at early follow up alters until the 1-year visit. Paired samples from 8 CD patients were examined but no separation between the time points could be detected by PCA (Fig. 4a) and no discrimination could be generated using OPLS-DA. Microbial temporal stability was further evaluated for the paired samples in relation to the additional (non-related) 13 samples from the 1-year follow up. First, a PCA analysis was generated to visualize within-patient mobility in the total population (Fig. 4b). Second, differences within and between patients were analyzed by Bray-Curtis dissimilarity and showed that the within-patient microbiota from early follow up to 1-year follow up was more similar than between-patient microbiota over time (Fig. 4c, two upper panels). Within-patient microbiota over time was also more similar than between-patient microbiota at the 1-year follow up (Fig. 4c, top and lower panel). No difference could be detected by Bray-Curtis dissimilarity between patients in endoscopic remission as compared to patients with disease recurrence at 1-year follow up (Fig. 4c, compare open and closed circles within each panel).

## Discussion

It is of clinical importance to identify CD patients at risk of disease recurrence after undergoing surgical resection. In this study, we demonstrate that the fecal microbiota composition of CD patients at 1-year follow up is associated with the postoperative disease status. Further, the microbial temporal stability within-patients was high during the first year after surgery. The analyses of fecal microbiota composition was performed by a user friendly test determining a selected set of bacteria, potentially allowing for microbiota profiles to be used as a diagnostic tool in a clinical setting to identify CD patients in need of more intense treatment regimens after surgery or vice versa, to identify patients at low risk of disease recurrence.

Similar to previous studies, we demonstrate that fecal microbiota profiles differed between CD patients and healthy subjects [7, 27]. The bacterial taxa discriminating CD patients from healthy subjects were found in lower abundance among patients and represented all of the four major bacterial phyla known to dominate the gut microbiota of humans, i.e., Firmicutes, Bacteroidetes, Actinobacteria and Proteobacteria [28, 29]. This suggests that CD is characterized by an impoverished gut microbiota, rather than a shift in abundance of specific species. The importance in using quantitative instead of relative abundance of bacteria has recently been appreciated and similar data have been obtained for CD patients using absolute abundance by estimating the 16S rRNA sequencing data to microbial load defined by flow cytometry [30] and fecal microbial DNA content [31].

Further, the fecal microbiota composition at the 1-year follow up diverged between CD patients who experienced postoperative disease recurrence and those who remained in remission, and a cluster analysis confirmed the relationship between the microbiota and the postoperative disease status. Among the bacterial taxa found to contribute to the separation between the two groups was *Alistipes*, found in higher abundance in patients who remained in remission as compared to patients suffering from subsequent disease recurrence. In a recent meta-analysis of 24 public studies, higher abundance of *Alistipes* was associated with a healthy state [32], likewise we showed that *Alistipes* is decreased in this cohort of CD patients as compared to healthy subjects.

Our results also suggest that the fecal microbiota composition of CD patients was subject to little variation during the first year after surgery. Thus, paired fecal samples from the same individual at early and 1-year follow up had similar microbiota composition, regardless of disease activity. Although fecal microbiota composition of IBD patients has been reported to fluctuate more than that of healthy subjects [7, 10], studies using GA-Map technology demonstrated low fecal microbiota variation over time in IBD patients undergoing anti-TNF therapy regardless of therapy outcome [33] as well as in healthy subjects [25]. The microbial temporal stability within patients together with the relatively strong association between fecal microbiota composition and postsurgical outcome indicates that fecal microbiota profiles may be used to predict disease course even shortly after surgery. Future studies should address whether microbiota profiles of stool samples, obtained before or in close conjunction to surgery indeed may be a biomarker for disease progression.

The GA-Map technology used in this study is a diagnostic tool previously validated for being able to discriminate IBD patients from healthy subjects, based on a selected set of fecal bacteria [25]. In comparison to deep-sequencing and shotgun metagenomic sequencing methods requiring advanced bioinformatics tools and reference datasets, the GA-Map technology allows the individual researcher/clinician to assess fecal microbiota composition without such expertise. Thus, the commercially available GA-map technology used in this study may allow for the realization of gut microbiota profiling as a diagnostic tool in a clinical setting and, in conjunction with the surgical procedure, may provide guidance for the identification of patients at risk of disease recurrence.

Even with its strengths and promising results, there are limitations and weaknesses with our study. The small number of study participants is a significant limitation to the generalizability of the study outcome, and might also have influenced the fit and predictive ability of microbiota profile analysis models. The limited size of the study also prevented us to do further analyses on subpopulations, e.g. with respect to various medical treatments and smoking among the patients. Further limitations are that we did not have access to fecal samples collected before the ileocaecal resection, and that fecal samples from the early follow up were obtained at a relatively broad time span, i.e. 3–10 weeks after resection. A strength, on the other hand, is that the paired fecal samples allowed us to address the microbial temporal stability in the CD patients. Results from these analyses indicated that the within-patient fecal microbiota composition was subject to little variation during the first year after surgery, regardless of disease activity. Thus, although an imminent risk that the perioperative antibiotic treatment would have disrupted the regular microbial flora for the patients at the early follow up, the bacteria detected by the GA-map dysbiosis test were still stable enough to display a similar profile over the first year after surgery.

## Conclusion

In summary, the validated GA-Map dysbiosis test allowed us to demonstrate that the fecal microbiota composition of CD patients is associated not only with the disease itself but also with the disease course 1 year after ileocecal resection. In addition, little variation in microbiota composition over time was seen within patients. Although larger studies are needed to confirm these findings our study suggests that fecal microbiota composition may be used as a non-invasive diagnostic tool for the postoperative disease course of CD patients.

## Supplementary information

**Additional file 1 **: **Table S1.** List of the 54 probes included in GA-map™ Dysbiosis Test; bacteria names and taxonomy and comparison of abundance between CD patients at 1-year follow up and healthy subjects (HS).

## Data Availability

The datasets used and/or analyzed during the current study are available from the corresponding author on reasonable request.

## Abbreviations

CD: Crohn’s disease
PCA: Principal component analysis
OPLS-DA: Orthogonal partial least squares discriminant analysis
VIP: Variable influence on projection
rRNA: Ribosomal RNA
IBD: Inflammatory bowel disease
