# Supplementary material for: Fecal microbiota composition is linked to the postoperative disease course in patients with Crohn’s disease
Source: BMC Gastroenterol. 2020 May 4;20:130. doi: 10.1186/s12876-020-01281-4 (PMC7197162; doi:10.1186/s12876-020-01281-4)
Supplement: Supplementary file 1 — Additional file 1 : Table S1. List of the 54 probes included in GA-map™ Dysbiosis Test; bacteria names and taxonomy and comparison of abundance between CD patients at 1-year follow up and healthy subjects (HS). [file 12876_2020_1281_MOESM1_ESM.docx]

| **Supplementary Table 1.** List of the 54 probes included in GA-map™ Dysbiosis Test; bacteria names and taxonomy and comparison of abundance between CD patients at 1-year follow up and healthy subjects (HS). | | | | |
| --- | --- | --- | --- | --- |
| **Genus/Species** | **Class** | **Phylum** | ***p-value*** | **Abundance in CD *vs*. HS** |
| Actinobacteria | Actinobacteria | Actinobacteria | 0.717 | - |
| Actinomycetales | Actinopbacteria | Actinobacteria | **0.010** | Decreased |
| *Atopobium rimae* | Corinobacteria | Actinobacteria | **0.043** | Decreased |
| *Bifidobacterium* spp. | Actinobacteria | Actinobacteria | 0.678 | **-** |
| *Alistipes* | Alistipes | Bacteroidetes | **< 0.0001** | Decreased |
| *Alistipes onderdonkii* | Alistipes | Bacteroidetes | **< 0.0001** | Decreased |
| *Bacteroides fragilis* | Bacteroides | Bacteroidetes | 0.104 | **-** |
| *Bacteroides pectinophilus* | Bacteroides | Bacteroidetes | **< 0.0001** | Decreased |
| *Bacteroide*s spp. | Bacteroides | Bacteroidetes | **0.002** | Decreased |
| *Bacteroides* spp. & *Prevotell*a spp. | Bacteroides | Bacteroidetes | **< 0.0001** | Decreased |
| *Bacteroides stercoris* | Bacteroides | Bacteroidetes | **0.006** | Decreased |
| *Bacteroides zoogleoformans* | Bacteroides | Bacteroidetes | **< 0.001** | Decreased |
| *Parabacteroides johnsonii* | Parabacterioides | Bacteroidetes | **< 0.001** | Decreased |
| *Parabacteroides* spp. | Parabacterioides | Bacteroidetes | **< 0.0001** | Decreased |
| *Prevotella nigrescens* | Prevotella | Bacteroidetes | **< 0.001** | Decreased |
| Firmicutes | Negativicutes/Clostridia | Firmicutes | **0.001** | Decreased |
| *Anaerotruncus colihominis* | Clostridia | Firmicutes | **< 0.0001** | Decreased |
| Bacilli | Bacilli | Firmicutes | 0.376 | **-** |
| *Bacillus megaterium* | Bacilli | Firmicutes | **0.005** | Decreased |
| *Catenibacterium mitsuokai* | Erysipelotrichia | Firmicutes | **0.001** | Decreased |
| Clostridia | Clostridia | Firmicutes | 0.321 | **-** |
| *Clostridium methylpentosum* | Ruminiclostridium | Firmicutes | **0.003** | Decreased |
| *Clostridium* sp. | Clostridia | Firmicutes | **0.005** | Decreased |
| *Coprobacillus cateniformis* | Erysipelotrichia | Firmicutes | **< 0.0001** | Decreased |
| *Desulfitispora alkaliphila* | Clostridia | Firmicutes | **< 0.001** | Decreased |
| *Dialister invisus* | Negativicutes | Firmicutes | 0.876 | **-** |
| *Dialister invisus* & *Megasphaera micronuciformis* | Negativicutes | Firmicutes | 0.876 | **-** |
| *Dorea* spp. | Clostridia | Firmicutes | 0.836 | **-** |
| *Eubacterium biforme* | Clostridia | Firmicutes | 0.071 | **-** |
| *Eubacterium hallii* | Clostridia | Firmicutes | 0.756 | **-** |
| *Eubacterium rectale* | Clostridia | Firmicutes | **0.017** | Decreased |
| *Eubacterium siraeum* | Clostridia | Firmicutes | **< 0.0001** | Decreased |
| *Faecalibacterium prausnitzii* | Clostridia | Firmicutes | **< 0.001** | Decreased |
| Lachnospiraceae | Clostridia | Firmicutes | 0.080 | **-** |
| *Lactobacillus ruminis* & *Pediococcus acidilactici* | Bacilli | Firmicutes | **0.005** | Decreased |
| *Lactobacillus* spp. | Bacilli | Firmicutes | 0.876 | **-** |
| *Lactobacillus* spp. 2 | Bacilli | Firmicutes | **< 0.0001** | Decreased |
| *Phascolarctobacterium* sp. | Negativicutes | Firmicutes | **< 0.0001** | Decreased |
| Ruminococcus albus & R. bromii | Clostridia | Firmicutes | 0.172 | **-** |
| *Ruminococcus gnavus* | Clostridia | Firmicutes | **0.048** | Increased |
| *Streptococcus agalactiae* & *Eubacterium rectale* | Bacilli | Firmicutes | 0.917 | **-** |
| *Streptococcus salivarius* ssp. *thermophilus* & S. *sanguinis* | Bacilli | Firmicutes | 0.640 | **-** |
| *Streptococcus salivarius* ssp.*thermophilus* | Bacilli | Firmicutes | 0.499 | **-** |
| *Streptococcus* spp. | Bacilli | Firmicutes | 0.272 | **-** |
| *Streptococcus* spp. 2 | Bacilli | Firmicutes | 0.208 | **-** |
| *Veillonella* spp. | Negativicutes | Firmicutes | 0.126 | **-** |
| Firmicutes (various) |  | Firmicutes/Tenericutes/Bacteroidetes species | **0.014** |  |
| Proteobacteria |  | Proteobacteria | 0.062 | **-** |
| *Acinetobacter junii* | Gammaproteobacteria | Proteobacteria | **0.033** | Decreased |
| Enterobacteriaceae | Gammaproteobacteria | Proteobacteria | **0.019** | Decreased |
| *Pseudomonas* spp. | Gammaproteobacteria | Proteobacteria | **< 0.001** | Decreased |
| *Shigella* spp. & *Escherichia* spp. | Gammaproteobacteria | Proteobacteria | **0.010** | Increased |
| *Mycoplasma hominis* | Mollicutes | Tenericutes | **< 0.0001** | Decreased |
| *Akkermansia muciniphila* | Verrumicrobiae | Verrucomicrobia | 0.126 | **-** |
